# Surfactant Protein-A inhibits *Aspergillus fumigatus*-induced allergic T-cell responses

**DOI:** 10.1186/1465-9921-6-97

**Published:** 2005-08-24

**Authors:** Seth Thomas Scanlon, Tatyana Milovanova, Sonja Kierstein, Yang Cao, Elena N Atochina, Yaniv Tomer, Scott J Russo, Michael F Beers, Angela Haczku

**Affiliations:** 1 Pulmonary, Allergy and Critical Care Division, Department of Medicine, University of Pennsylvania School of Medicine, Philadelphia, USA; 2 Institute for Environmental Medicine, University of Pennsylvania School of Medicine, Philadelphia, USA

## Abstract

**Background:**

The pulmonary surfactant protein (SP)-A has potent immunomodulatory activities but its role and regulation during allergic airway inflammation is unknown.

**Methods:**

We studied changes in SP-A expression in the bronchoalveolar lavage (BAL) using a murine model of single *Aspergillus fumigatus *(*Af*) challenge of sensitized animals.

**Results:**

SP-A protein levels in the BAL fluid showed a rapid, transient decline that reached the lowest values (25% of controls) 12 h after intranasal *Af *provocation of sensitized mice. Decrease of SP-A was associated with influx of inflammatory cells and increase of IL-4 and IL-5 mRNA and protein levels. Since levels of SP-A showed a significant negative correlation with these BAL cytokines (but not with IFN-γ), we hypothesized that SP-A exerts an inhibitory effect on Th2-type immune responses. To study this hypothesis, we used an *in vitro Af*-rechallenge model. *Af*-induced lymphocyte proliferation of cells isolated from sensitized mice was inhibited in a dose-dependent manner by addition of purified human SP-A (0.1–10 μg/ml). Flow cytometric studies on *Af*-stimulated lymphocytes indicated that the numbers of CD4+ (but not CD8+) T cells were significantly increased in the parental population and decreased in the third and fourth generation in the presence of SP-A. Further, addition of SP-A to the tissue culture inhibited *Af*-induced IL-4 and IL-5 production suggesting that SP-A directly suppressed allergen-stimulated CD4+ T cell function.

**Conclusion:**

We speculate that a transient lack of this lung collectin following allergen exposure of the airways may significantly contribute to the development of a T-cell dependent allergic immune response.

## Background

Surfactant protein (SP)-A belongs to a family of innate host defense proteins termed collectins because of the presence of **col**lagenous and also **lectin**-like domains [1]. SP-A has a potent immunomodulatory function with the N-terminal and C-terminal portions of the molecule capable of either activating or suppressing functions of cells of the immune system [2].

Allergen-induced asthma is characterized by the activation and steering of T-cells to a "helper" (Th) 2-type inflammation with eosinophilia, high serum immunoglobulin (Ig) E levels, reversible airway obstruction and hyper responsiveness (AHR) [3]. We have previously shown that allergic AHR is associated with disruption of surfactant biophysics, changes in the hydrophobic surfactant protein B and C [4] and upregulation of the lung collectin SP-D [5,6].

While SP-A is the most abundant and most extensively studied surfactant protein in the lung immune homeostasis [7-9], it is unclear how this molecule is regulated or functions during allergic inflammation. Published studies have shown that SP-A can inhibit lymphocyte function *in vitro *[10-12] and that administration of this protein in murine models ameliorated allergic changes in the lung [13-15], indicating a potential protective role. However, its direct regulatory action has not been detailed. The results of this study indicate specific changes in SP-A expression during development of an allergen induced immune response and show that SP-A can directly modulate the function of Th lymphocytes suggesting a regulatory link between innate and adaptive immunity in allergic airway inflammation.

## Methods

### Murine model of *Af *sensitization

Sensitized mice were injected intraperitoneally (i.p.) with 20 μg of *Af *(Hollister Stier, Elkhart, IN) and 20 mg Al(OH)_3 _(Imject Alum; Pierce, Rockford, IL) in PBS (100 μl) on days 1 and 14, followed by intranasal (i.n.) challenge on day 27 with 25 μl of allergen extract: (12.5 μg *Af *in 21% glycerol/ PBS). To analyze the kinetics of SP-A protein and mRNA changes BAL fluid and lung tissue were collected before (0 h) and 1, 6, 12, 24, 48 and 72 h after intranasal (i.n.) treatment, using a separate animal group at each time point (n = 6 in each) as described previously [4]. These time points were selected on the basis of our previous experience with acute models of locally elicited inflammation in mice [4,16,17]. *Af *sensitized and vehicle challenged mice (n = 8), non sensitized, *Af *challenged mice (n = 6) and non sensitized, glycerol challenged mice (n = 8) were studied 12 h after treatment. Naïve mice (n = 8) were used to control for the effects of i.p. sensitization and/or glycerol challenge. Lung function, BAL inflammatory cell and cytokine profile and SP-A levels were not significantly different between naïve animals and the groups that received sensitization plus glycerol challenge or glycerol challenge alone (not shown). All experimental subjects in this study were under a protocol approved by the Institutional Animal Care and Use Committee of the University of Pennsylvania.

### Analysis of SP-A protein and mRNA expression

Lungs were lavaged with sterile saline as previously described [4-6]. SDS-PAGE of the surfactant samples was carried out using NuPAGE 10% Bis-Tris gels (Novex, San Diego, CA). Western blots were performed using rabbit polyclonal anti-SP-A. Each lane was loaded with 10 μg of total protein. To investigate the absolute SP-A content in the BAL in addition to Western blot analysis, we used an ELISA protocol as described previously in detail [5]. Total RNA was isolated from lungs and specific SP-A mRNA content was determined by Northern blot analysis as described previously [6,18].

### Assessment of airway inflammation following intranasal challenge of *Af*-sensitized mice

*In vivo *measurement of lung function after *Af *challenge was performed in conscious, unrestrained, spontaneously breathing mice in a whole-body plethysmograph (Buxco Electronics Inc., Troy, NY) as described previously [4]. Airway function was quantified using the Enhanced Pause (Penh).

Analysis of total and differential cell count and cytokine profile of the BAL was carried out after lungs were lavaged with sterile saline as previously published [4,6,19]. Cytokine levels were measured in the cell free supernatant of the BAL by ELISA using antibodies and recombinant cytokines from PharMingen (San Diego, CA).

### Preparation of SP-A

**Figure 4:**
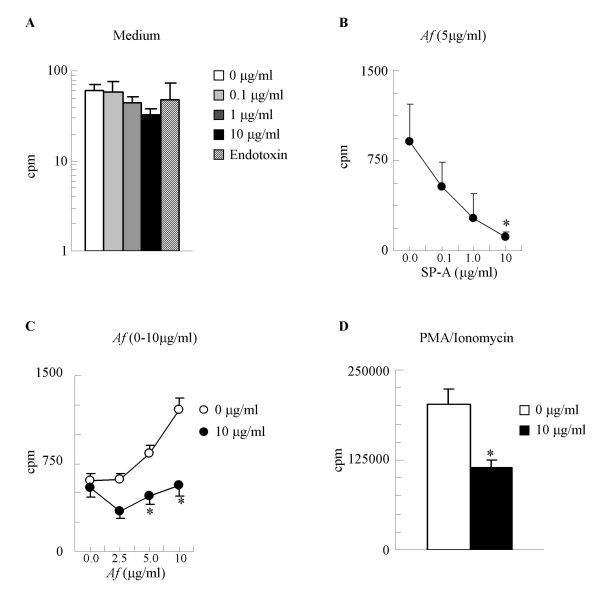
SP-A has a significant inhibitory effect on antigen specific lymphocyte proliferation from mice sensitized and challenged with *Af*. (A): Baseline (unstimulated) proliferation of splenic lymphocytes. Endotoxin in concentration equivalent to the endotoxin content of the 10 μg/ml SP-A samples did not affect cell proliferation. (B): *In vitro Af *stimulated proliferation of cells from mice sensitized and challenged with *Af *was inhibited by presence of SP-A, in a dose-dependent manner. (C): The *Af *dose-response curve of sensitized lymphocytes was abrogated in the presence of SP-A (10 μg/ml). (D) Presence of SP-A inhibited murine lymphocytes stimulated with PMA (2 ng/ml) and ionomycin (100 ng/ml). Background cpm (from wells that contain no cells): 0–16. Mean ± SEM of n = 4 independent experiments presented, each performed in triplicates. * p < 0.05: 0 *vs*. 10 μg/ml

Human SP-A was purified from human surfactant collected previously from patients with pulmonary alveolar proteinosis (PAP) as previously published [20]. The purified material was analyzed for protein concentration, subjected to Coomassie Blue staining (Figure 4A) and Western blot analysis (Figure 4B) and stored in aliquots at -80°C. The biological activity of the purified SP-A was tested in stimulated human peripheral blood monocytes (PBMC) (Figure 4C).

### Cell Proliferation Assays

Mice were sacrificed and spleens were harvested 24 hours after treatment. Spleens were homogenized in 10 mL of sterile PBS and layered over 5 mL of Histopaque (Histopaque-1077, Sigma) followed by density gradient centrifugation at 400 × g for 30 minutes at 18°C. Cells were then washed and cultured at 2 × 10^6 ^cells ml^-1 ^in U-bottom 96-well plates (Nunclon Delta Surface, Nunc, Denmark).

*Af *and PMA-Ionomycin (PI, Sigma-Aldrich, St. Louis, MO) were used as mitogens for the lymphocyte proliferation assay. Varying amounts of SP-A were added to stimulated lymphocytes. Mouse splenocyte cultures were incubated for 37°C in a humidified atmosphere with 5% CO_2 _for 96 h. At the 72 h time point, each well was pulsed with [^3^H] thymidine (0.2 mCi ml^-1^) for an additional 24 h at 37°C. Cells were then harvested on a PHD Harvester (Cambridge Technology, Cambridge, MA). [^3^H] thymidine incorporation was determined via liquid scintillation counting (Beckman Instruments, New York, NY). Samples were plated in triplicates and the experiments were repeated n = 4 times unless otherwise stated.

Human peripheral blood mononuclear cells were isolated from healthy volunteers using a standard protocol approved by the IRB of the University of Pennsylvania.

### Flow cytometric analysis of lymphocytes

The effects of SP-A on lymphocyte proliferation was further assessed using CFSE labeling as previously described [21]. Surface staining was performed at the time of harvest using Phycoerythin conjugated anti-mouse CD3, Fluorescin (FITC) conjugated anti-mouse CD4, FITC conjugated rat IgG_2b _isotype control (all from Pharmingen) and Allophycocyanin conjugated anti-human CD8 (Caltag, Burlingame, CA). Data were acquired on a four-color, dual laser FACSCalibur (Becton Dickinson, San Jose, CA). For FACS analysis the gated cell populations were divided into four different generations (G1, G2, G3 and G4) according to their CFSE content. Isotype controls were used to distinguish positive and negative populations. The SP-A-treated samples were compared with, and data were expressed as the percentage of the non-treated samples.

### Interleukin (IL)-4, IL-5 and IFN-γ assays

#### Real time PCR

A relative quantitation of lung IL-4, IL-5, and IFN-γ mRNA expression was performed by real time PCR using TaqMan(R) Gene Expression Assay and TaqMan(R) Universal PCR Master Mix: Mm00445259, Mm00439646, Mm00434204, and Mm00801778, respectively, (Applied Biosystems) according to the manufactures instructions. In brief three RNA samples isolated from the lungs per time points, were reverse transcribed using Superscript(TM) III First Strand Synthesis System (Invitrogen). Approximately 100 ng of cDNA was used per singleplex PCR reaction in a total volume of 25 μl. Cycling was performed on an ABI SDS-7000 and ABI SDS-7500 respectively with an initial denaturation step at 95°C for 10 min and 40 cycles of 15 sec. at 95°C and 1 min. at 60°C. Every sample was run in triplicate and β-2-microglobin (Mm00437762) was used as endogenous control. The Comparative Ct Method was used to analyze the results using ABI PRISM(R) 7900 SDS software. Results are expressed as fold difference (with range incorporating the standard deviation of the ddCt value into the fold difference calculation) relative to naïve animals.

#### *In vitro *release of cytokines from cultured lymphocytes

Splenic lymphocytes were cultured for 48 h as described. The supernatant was then removed and frozen at -80°C pending analysis. OptEIA mouse IL-4, IL-5 and IFN-γ ELISA kits (Pharmingen) were used.

### Data analysis

Data were expressed as mean ± SEM. Time courses and dose responses were compared using ANOVA. To test differences between individual groups Student's t test assuming equal variances were performed. Correlations were investigated by regression analysis. A p value of < 0.05 was considered as significant. Data were analyzed with the Sigma Stat standard statistical package (Jandel Scientific).

## Results

### Allergic sensitization and challenge of Balb/c mice with *Af *induced a transient fall in the levels of SP-A in the BAL fluid

**Figure 1:**
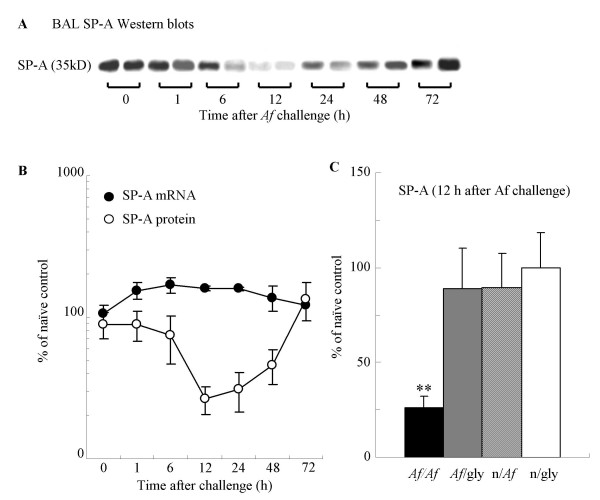
Allergen challenge of sensitized mice with *Af *induced a transient decrease in the BAL SP-A protein levels. (A) SP-A Western blots from two representative samples of BAL obtained 1, 6, 12, 24, 48 and 72 h after *Af *challenge. (B): SP-A protein from BAL (open circles) and lung tissue mRNA (closed circles); % of the naïve control values; n = 4–6. (C): SP-A protein levels measured 12 h after *Af *challenge Mean ± SEM of n = 8 each; ** p < 0.01 (*Af/Af *vs. *Af*/gly).

To study the relationship between changes in SP-A levels and the sequence of inflammatory events we used a model of single allergenic airway provocation [4]. Western blots were analyzed from the cell-free supernatant of the BAL fluid, using naïve control samples as standards that were run on each gel. Decreases in SP-A levels began at 1 h (88 ± 19% of naïve control) and reached the lowest values 12 h (26 ± 5%) after *Af *challenge. Recovery was observed 72 h later (135 ± 40 %) (Fig. 1A and 1B). To verify that the alterations in SP-A levels were specific at the 12 h time point, sensitized and *Af*-challenged mice (26.5 ± 5%) were compared with mice that received vehicle (glycerol) treatment instead of antigen (90.0 ± 19%; p < 0.05 n = 8, Fig 1C).

SP-A protein decreases were not associated with commensurate mRNA changes. Northern blot analysis on RNA extracted from the lung tissue of each group of sensitized mice showed that SP-A mRNA actually increased 12 h after allergen challenge in comparison with the 0 h controls (160 ± 6% *vs*. 106 ± 16 p < 0.05, n = 5, Fig 1B). The absolute BAL SP-A content (determined by ELISA) was decreased from 378 ± 46 to 239 ± 25 μg/lung (p < 0.05 n = 8) at 12 h and to 222 ± 22 μg/lung (p < 0.05 n = 8) 48 h after *Af *challenge.

### Decreased SP-A is associated with allergic airway inflammation to *Af *12 h after allergen provocation of sensitized mice

**Figure 2:**
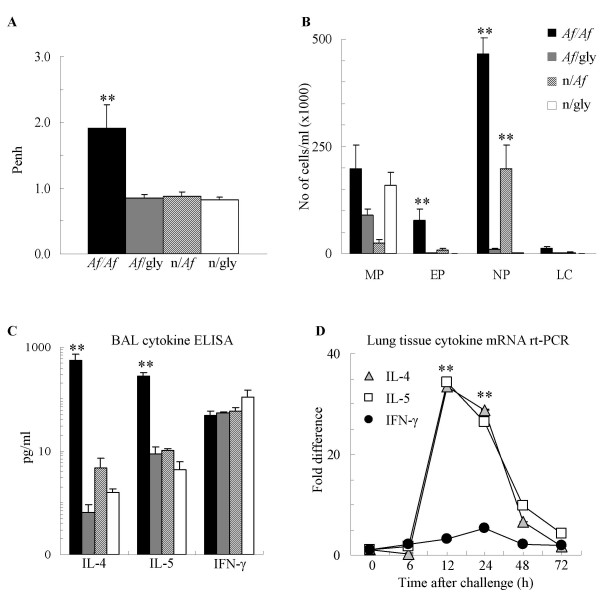
Single intranasal provocation of sensitized mice with *Af *induced increase in Penh, inflammatory cell influx and Th2 but not Th1 cytokine release, 12 h *Af*ter intranasal challenge. (A): Lung function measured by Penh. (B): BAL total and differential cell counts were used to calculate the absolute cell number. MP: macrophages EP: eosinophils, NP: neutrophils, LC: lymphocytes. (C): BAL Cytokine levels measured by ELISA (pg/ml). Mean ± SEM of n = 8 each; ** p < 0.01 (*Af/Af *vs. *Af*/gly) (D): Lung tissue cytokine mRNA levels measured by real-time PCR (fold increase in comparison to naïve control levels). ** p < 0.01 (vs. 0 h)

In order to examine the proinflammatory changes that were linked with the greatest fall in SP-A concentrations, we chose to study the 12 h time point. Following allergen provocation there were significant Penh increases 12 h after *Af*-challenge of sensitized mice (1.91 ± 0.36, n = 8) in comparison with glycerol challenge (0.88 ± 0.06, n = 8, p < 0.01). The glycerol challenge controls were not different from mice that received no sensitization or challenge (Fig 2A). Levels of Penh returned to near normal by 72 h (1.36 ± 0.1; n = 8). The cellular composition of BAL showed that 12 h after intranasal *Af *challenge there was a significant influx of inflammatory cells predominated by neutrophils (500 × 10^3 ^cells, p < 0.01, n = 8) and eosinophils (110 × 10^3 ^cells p < 0.01, n = 8) in comparison with glycerol challenged controls. These cell numbers were also reduced (1 ± 1 and 31 ± 19, respectively, n = 8) by the 72 h time point.

Similarly to our previously characterized ovalbumin and *Af*-induced models of allergic airway inflammation [4,6,19], i.p. sensitization and i.n. *Af *challenge induced a local production of predominantly Th2-type cytokines. In comparison with glycerol challenged controls there was a significant release of IL-4 (559 pg/ml *vs*. 3 pg/ml p < 0.01), IL-5 (283 pg/ml *vs*. 10 pg/ml p < 0.001; Figure 2C) and Eotaxin (173 pg/ml vs. 10 pg/ml p < 0.001) but not IFN-γ 12 h after allergen challenge (Figure 2C). Regression analysis using data pooled from the sensitized/*Af *challenged, sensitized glycerol challenged and non sensitized glycerol challenged mice revealed a significant, negative correlation with each of these cytokines (SP-A *vs*. IL-4: r = -0.61, p < 0.05; SP-A vs. IL-5: r = -0.82, p = 0.01; SP-A *vs*. Eotaxin: r = -0.75, p < 0.01). There was no correlation between SP-A and IFN-γ (not shown). Further, by the 72 h time point after allergen challenge IL-4 (8.7 ± 2.7 pg/ml), IL-5 (10.3 ± 7.3 pg/ml) and Eotaxin (9.72 ± 3.5 pg/ml) returned to close to baseline. To verify that the amount of airway SP-A is negatively associated with Th2 cytokine production, we also performed quantitative real-time PCR analysis of IL-4, IL-5 and IFN-γ mRNA. These results showed that mRNA levels for the Th2 cytokines increased approximately 35 fold in comparison with naïve controls 12 h after allergen challenge and returned to control levels by 72 h (Figure 2D).

### SP-A inhibits allergen-driven proliferation of lymphocytes *in vitro*

**Figure 3:**
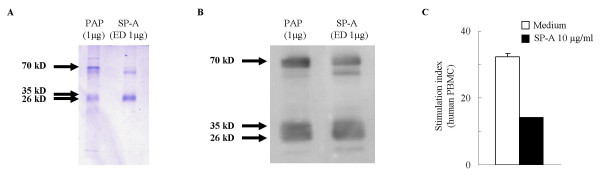
Purification and biological activity of SP-A from human PAP material. (A): Coomassie Blue staining. (B): Western blot comparison of endotoxin-depleted (ED) SP-A preparations (1 μg) with human PAP material (1 μg) used as a positive control. (C): Effect of purified human SP-A on normal human PBMC proliferation. Cells were stimulated with PMA (2 ng/ml) and ionomycin (100 ng/ml). Stimulation Index (cpm of stimulated cells divided by cpm of corresponding unstimulated cells). Mean ± SEM of n = 12

To test the hypothesis that SP-A exerts an inhibitory effect on Th2 processes, we used a model of *in vitro *allergen re-challenge of lymphocytes isolated from sensitized and control mice and treated the cells with SP-A purified from human PAP material. Figure 3 shows that following purification and endotoxin depletion the structural integrity (Fig 3A) and immunogenicity (Fig 3B) of SP-A remained intact. Purified, LPS-depleted human SP-A exhibited significant biological activity as assessed by its effects on proliferation of human PBMC (Fig 3C).

Splenic lymphocytes isolated from naïve mice and mice sensitized with *Af *were re-challenged *in vitro *with *Af *in the presence or absence of SP-A. ^3^H-thymidine uptake was expressed as cpm (counts per minute). The background cpm throughout our experiments ranged between 0 and 16. The SP-A preparation had < 0.07 endotoxin units/ml. This level is consistent with other cell culture SP-A preparations [10]. Furthermore, culturing murine lymphocytes in up to twice the amount of LPS (0.14 endotoxin units/ml) had no significant effect on lymphocyte function (Fig 4A). Baseline proliferation of naïve lymphocytes was not affected by SP-A (Figure 4A). It appears that an activated state of the lymphocytes is a prerequisite for inhibition by SP-A since sensitized cells stimulated with *Af *were markedly inhibited by SP-A in a dose-dependent manner (Fig 4B). Indeed greater antigenic stimulation resulted in greater suppression of proliferation by SP-A at 10 μg/ml (Fig 4C).

Suppression of T cell proliferation was not a result of sequestering *Af *in the culture because lymphocytes stimulated with PMA (10 ng/ml) and ionomycin (500 ng/ml) were inhibited by SP-A (10 μg/ml) to a similar extent as the *Af*-stimulated lymphocytes by 66 ± 4% (Figure 4D), suggesting a direct inhibitory action on activated cells.

### SP-A inhibits *Af*-induced CD3/CD4+ Th2 cell function

**Figure 5:**
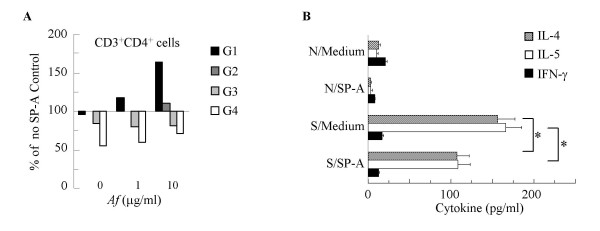
SP-A inhibited A*f*-induced CD4+ T cell proliferation and release of IL-4 and IL-5. (A): SP-A (10 μg/ml) increased the number of cells in the G1 (black bars) and decreased them in the G3-G4 populations of CD3/CD4+  lymphocytes (% change from the medium-treated samples). Average of 3 independent experiments. (B): *Af*-induced production of IL-4 and IL-5 by sensitized lymphocytes (S/Medium) was reduced in the presence of 10μg/ml of SP-A (S/SP-A) to levels similar to naïve lymphocytes (N/Medium).  Lymphocytes were stimulated by *Af* (1 μg/ml). IL-4 (grey bars), IL-5 (white bars) and IFN-γ (black bars). * p < 0.05 S/SP-A vs. S/Medium. Mean ± SEM of n = 4 experiments performed in triplicates..

To further confirm the effects of SP-A on the proliferating cells, we added an intracellular fluorescent dye 5,6-carboxy- fluorescein diacetate succinimidyl ester (CFSE) to the cell cultures. The amount of this dye is halved as the *Af*-stimulated lymphocytes divide, allowing individual generations to be detected using FACS analysis. Mononuclear cells were gated for positive CD3/CD4 or CD3/CD8 expression and marked as G1 (the highest CFSE content), G2, G3 and G4 population according to decreasing levels of CFSE expression. Cells incubated in the presence of 10 μg/ml of SP-A were compared with equivalent populations of cells stimulated in the absence of SP-A and the results are expressed as a percentage of the SP-A-free (100%) control. In comparison with medium treatment, (represented by the middle line) addition of SP-A increased the proportion of cells that remained in the G1 population in the samples stimulated by *Af *(Figure 5A). The proportion of the G1 cells in the SP-A treated CD3/CD4 populations were 95%, 118%, and 163% of the medium treated (SP-A-free) controls at 0, 1 and 10 μg/ml *Af *concentration, respectively. Thus, cells exposed to greater antigen stimulation appeared to be more susceptible to inhibition by SP-A. We did not observe a similar inhibitory effect by SP-A in the CD3/CD8 cells (not shown).

Addition of SP-A (10 μg/ml) to sensitized lymphocytes significantly inhibited IL-4 and IL-5 production both in the *Af*-induced (Figure 5B) and PMA/ionomycin stimulated (not shown) cultures (p < 0.05, n = 4). SP-A apparently also inhibited IFN-γ levels in the non-stimulated and in the PMA/ionomycin stimulated samples indicating a non-selective activity on cytokine production by stimulated T cells (not shown).

## Discussion

Emerging evidence suggests that collectins provide a link between innate and adaptive immunity. The relationship of SP-A production to inflammatory changes in the lung however is poorly defined. This study reports that a transient decrease in airway SP-A levels is associated with mRNA transcription and release of Th2-type cytokines following allergen provocation. The results also demonstrate that this lung collectin has a direct negative regulatory effect on allergen-induced lymphocyte activation *in vitro*.

Studies investigating allergen-induced airway inflammation previously described either significant increases [22,23] or decreases of this surfactant protein [24]. One reason for the conflicting data may be that levels of SP-A undergo transitional changes during development of allergic inflammation. Our time course study confirmed that levels of SP-A in the BAL dramatically decreased to 25% of the original 12 h after *Af *challenge but completely recovered 72 h later. The exact mechanisms that regulate the changes of SP-A concentrations in the airways are uncertain. Since reduction in the absolute amounts of SP-A was only 42%, the effect of dilution by the extravasated plasma proteins 12 h after allergen challenge could have significantly added to the fall in the fractional SP-A concentrations. Nevertheless, while further studies are needed to unravel its intricate regulation [25], it appears that an early decrease in the airway SP-A protein levels is not compensated immediately by enhanced synthesis or release, creating a relative, transient deficiency in this protein.

SP-A changes were comparable to that of SP-B and SP-C, described previously in a similar model [4]. Interestingly, while SP-B and SP-C protein levels in the BAL closely matched their respective mRNA recovered from the lung tissue, the fall SP-A protein was not paralleled by mRNA decreases. Thus (unlike in SP-B and SP-C), there is a dissociation between SP-A mRNA and protein regulation during the acute phase of an inflammatory response in the lung. Such discrepancy was also observed in a study looking at the effects of secretagogue-induced stimulation of SP-A synthesis [26] and in an *in vitro *model where SP-A mRNA but not protein expression was induced by dexamethasone, cyclic AMP and IL-4 [27]. In addition to not promptly following changes in mRNA production, SP-A did not require constitutive protein synthesis indicating a long intracellular half life [27]. Taken together, our previous and current work suggests that there is a significant lag between transcriptional, translational and/or secretory processes in SP-A regulation. Although the implications of such SP-A deficiency are unclear, the regulatory effects on the adaptive immune function during development of an inflammatory response could be significant.

The inverse association we observed between levels of SP-A and the inflammatory changes 12 h after allergen provocation was used to generate our hypothesis that SP-A negatively regulates Th2 cytokine production. This hypothesis was then tested by directly adding SP-A to allergen-stimulated T cells. These results are in support of previous reports showing that administration of SP-A alleviated allergic inflammatory changes in mice (reviewed in [15]), however the underlying mechanisms remain unclear. Binding of inhaled allergens such as particles of *Af *[28,29] and house-dust mite [30] may be one way to interfere with the allergic response. A second mechanism could be an interference with the function of inflammatory effector cells as indicated by inhibitory effects of SP-A on chemokine release by activated eosinophils [13,31]. Further, recent research has highlighted the role of SP-A on adaptive immune functions such as antigen presentation by dendritic cells [32] and T cell activation [10-12]. SP-A inhibited T-cell proliferation induced by various non-specific stimulators through both an IL-2 independent and an IL-2 and T cell receptor dependent manner [12].

The mechanism of T cell susceptibility to inhibition by SP-A needs further clarifications. While the inhibitory effects of SP-A have been demonstrated in antigen-stimulated human PBMC [33] and more recently, in a murine system of splenocytes co-cultured with ovalbumin-specific T cell hybridomas [12], no study has examined the effect of SP-A on T cell proliferation in a mouse model of allergen-induced asthma *in vitro*. Here we present for the first time the inhibitory effects of SP-A on generational progression of sensitized lymphocytes to *Af *re-challenge *in vitro*. We show that this inhibition was dose-dependent. Further, inhibition of lymphocyte activation by SP-A was not due to toxic effects since baseline proliferation of the lymphocytes was not affected. In addition, Figure 5A demonstrates that CD3+/CD4+ lymphocytes had significantly inhibited proliferative ability (as measured by the CFSE content of the cells) in the presence of SP-A. In this FACS analysis study the dead cells were excluded and only the live cells were analyzed (an equal number of 10,000 events collected in every sample). These results suggest that SP-A has a direct effect in suppressing proliferation of cells. Previous studies similarly showed that neither SP-A nor SP-D affects cell viability or apoptosis in PMA and ionomycin-stimulated cells [12]. Similarly to a study using cells from human asthmatics [33], we also showed that the inhibitory effect of SP-A did not depend on interference with the antigen-T cell receptor interaction since it suppressed both antigen- and mitogen-stimulated models of proliferation. Our studies however further indicated that in antigen-stimulated cultures sensitized cells exposed to greater antigen concentration and thus induced to proliferate more vigorously, were more susceptible to inhibition by SP-A. In addition, in the presence of SP-A there were significantly more sensitized CD4+ (not CD8+) lymphocytes in the parental (G1) population at higher *Af *concentrations, confirming that the antigen-responsive, stimulated T helper cell population is the target of SP-A.

The effects of SP-A treatment on inhibiting Th2 cytokines were previously studied *ex vivo *[34]. Here we show that greater amount of airway SP-A was associated with a lesser extent of Th2 cytokine release *in vivo *and that there was a significant, negative correlation between SP-A and the amount of IL-4, IL-5 and eotaxin supporting the hypothesis that this collectin exerts an inhibitory effect on Th2 processes. Indeed addition of SP-A abolished production of the Th2 cytokines IL-4 and IL-5 by *Af*-stimulated lymphocytes. Thus, SP-A may act as a natural immunosuppressant in the lung with direct inhibitory actions on allergen-stimulated CD4+ Th2 lymphocytes.

## Conclusion

A transient decrease in airway levels of SP-A was associated with transcription of mRNA and release of the Th2-type cytokines IL-4 and IL-5 but not IFN-γ. On the other hand, addition of SP-A to lymphocyte cultures from sensitized mice suppressed allergen-induced Th2-type immune response. We speculate that this lung collectin may play a protective role to prevent allergen-inhalation provoked immune reactions and propose the novel concept that a transient SP-A deficiency contributes to the pathogenesis of the allergic airway response.

## List of abbreviations

*Af*: Aspergillus fumigatus

AHR: Airway hyperresponsiveness

BAL: Bronchoalveolar lavage

CFSE: 5,6-carboxyfluorescein diacetate succinimidyl ester

cpm: Count per minute

ELISA: Enzyme linked immunosorbent assay

FACS: Fluorescence activated cell sorter

FITC: Fluorescein isothiocyanate

Ig: Immunoglobulin

IL: Interleukin

i.n.: Intranasal

i.p.: Intraperitoneal

LPS: Lipopolysaccharide

MBL: Mannose binding lectin

SP: Surfactant protein

PAP: Pulmonary Alveolar Proteinosis

PI: PMA and ionomycin

PMA: Phorbol 12-myristate 13-acetate

PBMC: Peripheral Blood Mononuclear Cells

Th: T helper

## Authors' contributions

Seth Thomas Scanlon carried out the surfactant protein analysis and the protein and phospholipids measurements. Tatyana Milovanova carried out the CFSE studies and FACS analysis. Sonja Kierstein performed the cytokine mRNA analysis. Yang Cao participated in the surfactant protein analysis and in the animal experiments. Elena N. Atochina analyzed the SP-A Western and Northern blots. Yaniv Tomer carried out the animal experiments. Scott J. Russo performed the mRNA extraction and Northern blots. Michael F. Beers participated in the design of the study and contributed to the manuscript writing with comments. Angela Haczku conceived of, designed and coordinated the study, and participated in its writing. All authors approved the manuscript.
